# Short-term clinical outcomes of laparoscopic duodenum-preserving pancreatic head resection for the management of pancreatic-head cystic neoplasms

**DOI:** 10.1186/s12893-023-01985-w

**Published:** 2023-04-28

**Authors:** Zhaozhi Xia, Shuchao Zhao, Xin Gao, Hongrui Sun, Faji Yang, Huaqiang Zhu, Hengjun Gao, Jun Lu, Xu Zhou

**Affiliations:** Department of Hepatobiliary Surgery, Shandong Provincial Hospital Affiliated to Shandong First Medical University, No. 324, Jingwuweiseven Road, Huaiyin District, Jinan, China

**Keywords:** Duodenum-Preserving pancreatic Head Resection, Pancreaticoduodenectomy, Pancreatic-head cystic Neoplasms, Short-term outcomes, Laparoscopy

## Abstract

**Background:**

In this study, we aimed to investigate the short-term clinical outcomes of laparoscopic duodenum-preserving pancreatic-head resection (LDPPHR) for the management of pancreatic-head cystic neoplasms.

**Methods:**

This retrospective study included 60 patients who were treated with pancreatic-head cystic neoplasms at the Shandong Provincial Hospital Affiliated to Shandong First Medical University from December 2019 to July 2022.

**Results:**

No significant difference was found between the two groups in terms of the baseline and pathological characteristics of patients (*P* > 0.05). The postoperative exhaust time was shorter in the LDPPHR group compared with the laparoscopic pancreaticoduodenectomy (LPD) group (2 (2 and 4) vs. 4 (3 and 5) days; *P* = 0.003). No significant difference was found between the two groups in terms of operative time, estimated blood loss, intraoperative transfusion, hemoglobin levels on the first postoperative day, total bilirubin before discharge, direct bilirubin before discharge, postoperative hospital stay, postoperative pancreatic fistula, bile leakage, hemorrhage, peritoneal effusion, abdominal infection, delayed gastric emptying, interventional embolization hemostasis, reoperation, and 30-day readmission (*P* > 0.05). No conversion and 90-day mortality were found in the two groups. The LDPPHR group showed a higher 3-month postoperative PNI, 6-month postoperative TG and 6-month postoperative BMI than the LPD group (*P* < 0.05).

**Conclusions:**

Compared with LPD, LDPPHR can decrease the postoperative exhaust time of patients, improve the short-term postoperative nutritional status, and does not decrease the safety of the perioperative period.

## Introduction

The increased awareness of health examination among people and the advancement of modern diagnostic imaging technology has increased the detection rate of pancreatic cystic tumors. Pancreatic cysts are found in almost 2–45% of the population [1]. The detection of pancreatic cysts by MRI is almost 13.5%[2]. Pancreatic cysts are classified as non-neoplastic and neoplastic cysts. Neoplastic pancreatic cysts, also known as pancreatic cystic tumors, mainly include serous cystadenoma, mucinous cystic tumors, intraductal papillary mucinous tumors, solid pseudopapillary tumors, and neuroendocrine tumors [3, 4]. Pancreatic cystic tumors are potentially malignant, and surgical resection is the best treatment strategy [5]. Pancreaticoduodenectomy (PD) is considered the standard treatment procedure for cystic tumors present on the pancreatic head. Duodenum-preserving pancreatic head resection (DPPHR) was initially performed on inflammatory masses found in the head of the pancreas [6, 7], and its application has been extended to treat cystic tumors present on the head of the pancreas [8–10]. Compared with PD, DPPHR preserves the continuity of the gastroduodenum and the exocrine and exocrine functions of the pancreas [11–13]. With the development of laparoscopic technology, laparoscopic pancreaticoduodenectomy (LPD) is also advancing, and the wide application of LPD in major medical centers has promoted the development and generalization of laparoscopic duodenum-preserving pancreatic head resection (LDPPHR). In this study, we retrospectively analyzed the clinical data of 60 patients with cystic tumors of the pancreatic head admitted at the Shandong Provincial Hospital Affiliated to Shandong First Medical University from December 2019 to July 2022. We aimed to investigate the short-term clinical efficacy of LDPPHR for treating pancreatic head cystic tumors.

## Materials and methods

### Patients

The clinical data of 60 patients who were treated for pancreatic-head cystic neoplasms at the Shandong Provincial Hospital Affiliated to Shandong First Medical University from December 2019 to July 2022 was collected. Of these 60 patients, 31 had undergone LDPPHR and 29 had undergone laparoscopic pancreaticoduodenectomy (LPD). The subject inclusion criteria were as follows: (1) combined with a preoperative medical history, tumor markers, imaging, and postoperative pathological diagnosis of pancreatic head cystic tumor, including serous cystic adenoma (SCA), mucinous cystic neoplasm (MCN), intraductal papillary mucinous neoplasm (IPMN), solid pseudopapillary tumor (SPN), and pancreatic neuroendocrine neoplasm (PNET, G1/G2); (2) patients with normal preoperative cardiopulmonary function and hence could withstand surgery; (3) patients without any other serious underlying medical conditions that were concurrent; (4) complete clinical data. The patient exclusion criteria were as follows: (1) age ≥ 80 years; (2) intraoperative and postoperative pathological diagnosis indicating malignancy; (3) incomplete clinical data. This study was approved by the Medical Ethics Committee of the Provincial Hospital Affiliated to Shandong First Medical University.

### Surgical techniques

#### LDPPHR

**Fig. 1:**
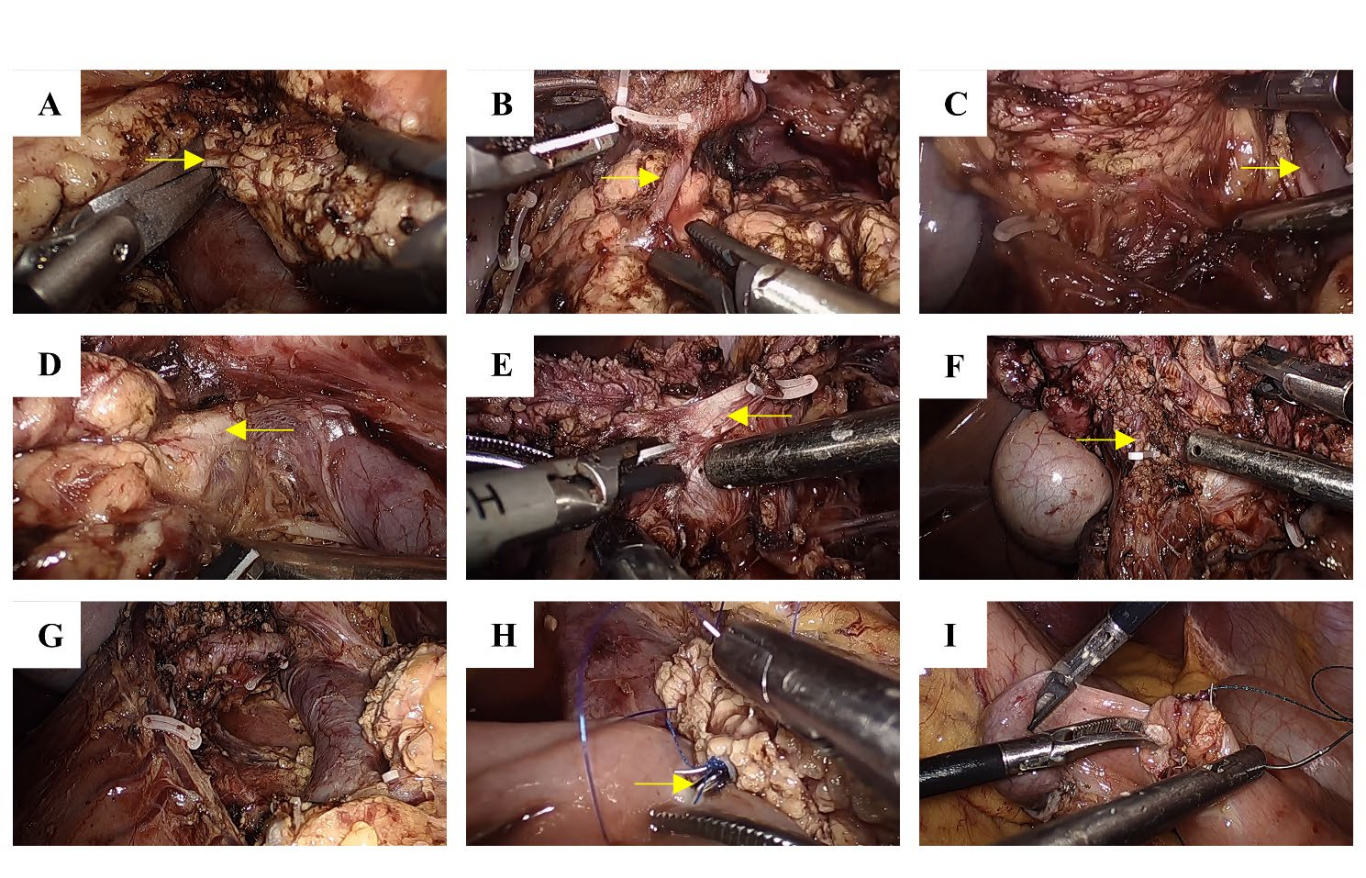
The surgical field. (**A**) The retropancreatic tunnel was established, and the main pancreatic duct was sharply excised. The yellow shear indicates the pancreatic duct. (**B**) The ASPDA was isolated along the GDA for amputation. The yellow shear indicates ASPDA.(**C**) The uncinate process of the pancreas was excised from the foot side to the cephalad side of the duodenum using an ultrasonic scalpel. The yellow shear indicates SMV. (**D**) Intraoperative discovery of the common bile duct. The yellow shear indicates the bile duct. (**E**) The head of the pancreas was excised from the head to the foot along the left side of the common bile duct. The yellow shear indicates the bile duct. (**F**) Clipping of the main pancreatic duct in the ampulla. The yellow shear indicates the pancreatic duct. (**G**) The surgical field after pancreatic head resection. (**H**) “Bing” R8 pancreaticojejunostomy. The yellow shear indicates the main pancreatic duct stent catheter. (**I**) Jejuno–jejunum side-to-side anastomosis

Of the 60 patients enrolled in this study, 31 underwent three-dimensional reconstruction before surgery. For this procedure, the patient was placed in the supine position, with the head high and the feet lowered by approximately 30°. Trocar puncture was then performed by following the conventional 5-hole method. The observation hole (10 mm) was located under the umbilicus, with two 12-mm holes on the right side, one 12-mm hole and one 5-mm hole on the left side (the left and right sides were symmetrically distributed in the midclavicular line and axilla frontline. The entire abdominal cavity was explored to rule out abdominal tumor metastasis. The gastrocolic ligament was opened, the right gastroepiploic blood vessel was severed, and the pancreatic head and pancreatic neck tissues were fully exposed. The lymph nodes of group 8a were dissected and sent for intraoperative frozen pathological examination. The anterior superior pancreaticoduodenal artery (ASPDA) was isolated along the gastroduodenal artery (GDA) for amputation. The superior mesenteric vein (SMV) was searched at the lower border of the pancreas, a retro-pancreatic tunnel was established, and the neck of the pancreas was severed with an ultrasonic scalpel. The pancreatic head and pancreatic neck were turned to the right, and the uncinate process of the pancreas was excised from the foot side to the cephalad side of the duodenum with an ultrasonic scalpel. When the resection reached the descending segment of the duodenum, the common bile duct was carefully separated and identified at the upper end of the head of the pancreas, while the head of the pancreas was removed from the head to the foot along the left side of the common bile duct and the tumor specimen was completely removed. The specimens were removed through a 3–5-cm incision in the midline of the upper abdomen and sent for intraoperative rapid freezing for the subsequent pathological examination. The severed end of the main pancreatic duct in the ampulla was sutured and closed. Next, the pancreatic duct support tube was placed, and the jejunum was severed at a distance of 15 cm from the ligament of Trevor. Pancreaticojejunostomy was performed using the “Bing” R8 anastomosis method [14]. The jejunal anastomosis was performed at a distance of 50 cm from the pancreaticojejunostomy, the mesenteric hiatus was closed, and the abdominal cavity was washed repeatedly to ensure the absence of any active bleeding. Two abdominal irrigation and drainage tube were placed on the anterior and posterior walls of the pancreaticojejunostomy, and the operation was completed. The surgical field is illustrated in Fig. 1.

#### LPD

Of the 60 patients, 29 underwent LPD. In this procedure, the surgical position and the trocar layout were kept the same as those in the LDPPHR group. The entire abdominal cavity was explored to exclude tumor metastasis from the abdominal cavity, and the stomach was cut 4 cm away from the pylorus. The gallbladder was removed, the hepatic duct was severed, the retropancreatic tunnel was established, and the neck of the pancreas was severed with an ultrasonic scalpel. The duodenum was dissociated via Kocher’s maneuver, the jejunum was severed at a distance of 10 cm from the ligament of Trevor, the proximal end of the jejunum was lifted, the uncinate process of the pancreas was excised from the foot side to the head with an ultrasonic scalpel, and the blood vessels from the superior mesenteric artery (SMA) to the uncinate process of the pancreas were ligated and removed. Complete resection of the surgical specimens was performed, followed by pancreaticoenteric anastomosis (same as for LDPPHR), bileoenteric anastomosis, and gastrointestinal anastomosis. The abdominal cavity was washed repeatedly to confirm the absence of any active bleeding. One abdominal cavity irrigation and a drainage tube were placed on the anterior and posterior walls of the pancreaticoenteric anastomosis and the posterior wall of the biliary-enteric anastomosis, and then the operation was completed.

### Data collection

(1) Preoperative and intraoperative conditions: The baseline and pathological characteristics of the patients, operation time, estimated blood loss, intraoperative transfusion, and conversion to laparotomy. Relevant indicators refer to the anesthesia record sheet. (2) Postoperative conditions: hemoglobin on the first postoperative day, total bilirubin before discharge, direct bilirubin before discharge, postoperative exhaust time, postoperative hospital stay, reoperation, interventional embolization, 30-days readmission, and 90-day mortality. (3) Short-term postoperative complications: pancreatic fistula, biliary fistula, hemorrhage, peritoneal effusion, intra-abdominal infection, and delayed gastric emptying. Relevant definitions refer to the diagnostic criteria as proposed by the International Surgery Group. (4) Postoperative short-term nutritional indicators: 3-month postoperative PNI, 6-month postoperative PNI, 3-month postoperative TCH, 6-month postoperative TCH, 3-month postoperative TG, 6-month postoperative TG, 3-month postoperative BMI, and 6-month postoperative BMI. In this study, “short-term” was defined as the period between the postoperative period and the discharge of the patient within 6 months.

### Statistical analysis

SPSS 22.0 statistical software was used for statistical analysis. The measurement date with a normal distribution was described as x ± s, and the t-test was applied for intergroup comparisons. Measurement data with skewed distribution were described as M (*P* 25, *P* 75), and the Mann–Whitney U-test was performed for intergroup comparisons. The counting data were described as a percentage, and the χ2 test or Fisher’s exact test was performed for intergroup comparisons. *P* < 0.05 was considered to indicate statistical significance.

## Results

### Baseline and pathological characteristics of patients

No significant difference was found between the two groups in terms of age, gender, preoperative comorbidities, abdominal surgery history, BMI, American Society of Anesthesiologists (ASA) score, preoperative hemoglobin, preoperative total bilirubin, preoperative direct bilirubin, preoperative albumin, postoperative CA19-9 levels, and pathological diagnosis and lesion diameter (*P* > 0.05). Baseline characteristics and pathological outcomes are presented in Table 1.

**Table 1:** Baseline characteristics and pathological outcomes of patients. BMI - body mass index; ASA - American Society of Anesthesiologists; CA19-9 - carbohydrate antigen 19 − 9; SCA - serous cystic adenoma; MCN - mucinous cystic neoplasm; SPN - solid pseudopapillary tumor; IPMN - intraductal papillary mucinous neoplasm; PNET - pancreatic neuroendocrine neoplasm

Parameter	LDPPHR group	LPD group	*P* value
	(n = 31)	(n = 29)	
Age (years)	48.4 ± 15.8	52.3 ± 11.9	0.29
Gender (n, %)			
male	8 (25.8%)	14 (48.3%)	0.07
female	23 (74.2%)	15 (51.7%)	
Preoperative comorbidities (n, %)			
hypertension	7 (22.6%)	4 (13.8%)	0.51
diabetes	3 (9.7%)	2(6.9%)	1.00
anemia	2 (6.5%)	0	0.49
Bronchial Asthma	1 (3.2%)	0	1.00
Abdominal surgery history (n, %)	4(12.9%)	6(20.7%)	1.00
BMI (kg/m^2^)	24.9 ± 4.0	23.9 ± 2.9	0.26
ASA (n, %)			
I	16 (51.6%)	19 (65.5%)	0.64
II	14 (45.2%)	9 (31.0%)	
III	1 (3.2%)	1 (3.4%)	
Preoperative hemoglobin (g/L)	127.6 ± 18.2	133.7 ± 16.3	0.18
Preoperative total bilirubin (mmol/L)	11.4 (8.1,12.9)	12.4 (10.1,16.5)	0.06
Preoperative direct bilirubin (mmol/L)	2.1 (1.5,2.7)	2.3 (1.8,3.1)	0.10
Preoperative albumin (g/L)	40.1 ± 2.9	40.8 ± 4.1	0.41
Preoperative CA19-9 (IU/mL)	9.7 (7.1,13.4)	11.1 (6.0,16.5)	0.89
Pathological diagnosis			
SCA	9 (29.0%)	4(13.8%)	0.16
MCN	1(3.2%)	6(20.7%)	
SPN	7(22.6%)	8(27.6%)	
IPMN	8(25.8%)	8(27.6%)	
PENT(G1/G2)	5(16.1%)	2(6.9%)	
Vesicular transformation	1(3.2%)	0	
Benign cystic lesions	0	1(3.4%)	
Lesion diameter (cm)	2.8 (1.6,5.0)	3.5 (2.5,4.7)	0.32

### Short‑term surgical outcomes of patients

The postoperative exhaust time was lesser in the LDPPHR group compared with the LPD group (2 (2 and 4) vs. 4 (3 and 5) days; *P* = 0.003). No significant difference was found between the two groups in terms of operative time, estimated blood loss, intraoperative transfusion, hemoglobin on the first postoperative day, total bilirubin before discharge, direct bilirubin before discharge, postoperative hospital stay, postoperative pancreatic fistula, bile leakage, hemorrhage, peritoneal effusion, abdominal infection, delayed gastric emptying, interventional embolization hemostasis, reoperation, and 30-day readmission (*P* > 0.05). No conversion and 90-day mortality were found in the two groups. Short‑term surgical outcomes of patients are presented in Table 2.

**Table 2:** Short-term surgical outcomes of two groups

Parameter	LDPPHR group	LPD group	*P* value
	(n = 31)	(n = 29)	
Operative time (min)	315 (250, 445)	350 (270, 390)	0.50
EBL (ml)	100 (50, 200)	100 (50, 150)	0.77
Intraoperative transfusion (n, %)	4 (12.9%)	0	0.11
Conversion (n, %)	0	0	
Hemoglobin on the first postoperative day (g/L)	121.8 ± 17.2	119.5 ± 17.3	0.60
Total bilirubin before discharge (mmol/L)	13.5 (9.7, 19.0)	13.1 (11.0, 19.8)	0.86
Direct bilirubin before discharge (mmol/L)	3.5 (2.3, 5.5)	4.2 (2.7, 7.4)	0.23
Postoperative exhaust time (Days)	2 (2, 4)	4 (3, 5)	**0.003**
Postoperative hospital stay (Days)	11 (8, 18)	11 (8, 17)	0.80
Postoperative pancreatic fistula (n, %)			
non/biochemical leakage	22 (71.0%)	22 (75.9%)	1.00
grade B	8 (25.8%)	7 (24.1%)	
grade C	1 (3.2%)	0	
Bile leakage (n, %)	3 (9.7%)	5 (17.2%)	0.47
Hemorrhage (n, %)	3 (9.7%)	1 (3.4%)	0.61
Peritoneal effusion (n, %)	4 (12.9%)	3 (10.3%)	0.54
Abdominal infection (n, %)	2 (6.5%)	3 (10.3%)	0.67
DGE (n, %)	3 (9.7%)	4 (13.8%)	0.70
Interventional embolization hemostasis (n, %)	2 (6.5%)	1 (3.4%)	1.00
Reoperation (n, %)	0	0	
30-day readmission (n, %)	1 (3.2%)	1 (3.4%)	1.00
90-day Mortality (n, %)	0	0	

EBL - estimated blood loss; DGE - delayed gastric emptying

### Postoperative short-term nutritional indicators of two groups

The LDPPHR group showed a higher 3-month postoperative PNI, 6-month postoperative TG, and 6-month postoperative BMI than the LPD group (*P* < 0.05). No significant difference was found between the two groups in terms of their 6-month postoperative PNI, 3-month postoperative TCH, 6-month postoperative TCH, 3-month postoperative TG ,and 3-month postoperative BMI (*P* > 0.05). Postoperative short-term nutritional indicators of the study patients are presented in Table 3.

**Table 3:** Postoperative short-term nutritional indicators of two groups

Parameter	LDPPHR group	LPD group	*P* value
	(n = 31)	(n = 29)	
PNI (3 months postoperatively)	49.5 ± 1.9	47.4 ± 2.5	**0.001**
PNI (6 months postoperatively)	50.1 ± 1.8	49.3 ± 2.9	0.21
TCH (3 months postoperatively, mmol/L)	4.57 ± 0.34	4.46 ± 0.26	0.18
TCH (6 months postoperatively, mmol/L)	4.80 ± 0.35	4.63 ± 0.28	0.05
TG (3 months postoperatively, mmol/L)	1.51 ± 0.20	1.47 ± 0.18	0.34
TG (6 months postoperatively, mmol/L)	1.63 ± 0.24	1.48 ± 0.21	**0.01**
BMI (3 months postoperatively, kg/m^2^)	24.3 ± 2.5	23.1 ± 2.8	0.12
BMI (6 months postoperatively, kg/m^2^)	24.8 ± 2.1	23.3 ± 2.6	**0.02**

PNI - prognostic nutrition index; TCH - total cholesterol; TG - triglyceride; BMI - body mass index

## Discussion

Presently, only a few studies have reported LDPPHR based on surgical experience and case reports. In 2016, Zhou et al. [15] reported the first case of LDPPHR for treating a solid pseudopapillary tumor present on the pancreatic head. In 2019, Cao et al. [16] reported 12 patients who underwent LDPPHR for the treatment of benign and low-grade malignant tumors of the pancreatic head, which opened up a minimally invasive way for LDPPHR for treating benign or low-grade malignant tumors of the pancreatic head. In this study, we found that the postoperative exhaust time in the LDPPHR group was less compared with the LPD group, and the difference was statistically significant. This indicated that LDPPHR can preserve the integrity of the duodenum and biliary tract compared with the results obtained by LPD; therefore, the recovery of postoperative gastrointestinal function of patients is lesser. Postoperative exhaust time is an indicator of a patient’s postoperative recovery, and the early appearance of postoperative exhaust helps reduce the discomfort of bloating as well as alleviates the negative emotions arising during hospitalization [17]. In addition, we found that the LDPPHR group had a higher 3-month postoperative PNI, 6-month postoperative TG ,and 6-month postoperative BMI than the LPD group (*P* < 0.05). Witzigmann et al. [18] reported that the postoperative BMI in the DPPHR group was significantly higher than that in the classic PD group (*P* < 0.001), which is consistent with our study. Comparatively, LDPPHR offers the advantage of postoperative nutritional recovery. The study found that the cause of NAFLD after PD was malnutrition [19]. Kato et al. [20] reported that the serum albumin level in the DPPHR group were significantly better than that in the PD group (4.2 vs. 3.9; *P* = 0.003), the prognostic nutritional index of the DPPHR group was better (albeit the difference was not significant), and the incidence of postoperative NAFLD in the DPPHR group was also lower than that in the PD group. As LDPPHR can better maintain the continuity of the stomach-duodenum-bile duct, LDPPHR offers more advantages than LPD in terms of postoperative gastrointestinal function recovery and nutritional status.

The anatomy of the blood vessels in the pancreaticoduodenal region is complex. The posterior superior pancreaticoduodenal artery (PSPDA) and the posterior inferior pancreaticoduodenal artery (PIPDA) comprise the posterior pancreaticoduodenal arterial arch [21, 22], and the complete preservation of the posterior arteriolux of the pancreatic duodenum is crucial to ensure sufficient blood supply to the duodenum and common bile ducts [23]. Postoperative bleeding occurred in 3 patients in the LDPPHR group, of which two underwent celiac arteriography embolization successfully. Intraoperative angiography showed that the bleeding vessel was the stump of the ASPDA. We usually choose to cut off the ASPDA during LDPPHR surgery to decrease the risk of poor exposure in the surgical field. It can be more difficult for the GDA stump in LPD to withstand the corrosion of pancreatic juice. If a pancreatic fistula occurs post-surgery and the drainage is not smooth, the stump will bleed under the erosion of pancreatic juice. Preventing the rupture of the end of ASPDA is also a major concern.

Owing to the special course and anatomical position of the common bile duct and the pancreas during LDPPHR, its exposure and protection are also the keys and difficult points of the surgery, and the corresponding common bile duct injury can occur occasionally. Chen et al. [24] proposed the concept of the common bile duct triangle. After the pancreatic neck is severed, the pancreatic head is pulled to right by 90°, and the triangle formed by the left border of the GDA, the right border of the portal vein, and the upper border of the pancreas can be common, and the bile duct can be detected. The application of real-time indocyanine green fluorescence imaging in endoscopic surgery can decrease the risk of intraoperative common bile duct injury [25–27]. PSPDA runs forward through the distal bile duct, descends along the right border of the bile duct, and re-passes at the level of the posterior papilla [8]. Excessive separation of pancreatic tissue between the bile ducts and duodenum during LDPPHR can damage the posterior artery of the superior duodenum, which affects the blood flow of the bile ducts and duodenum. We routinely performed 3D reconstruction before surgery to facilitate the intraoperative assessment of the spatial relationship between the lesion area and the common bile duct. During the surgery, the surgical team tries to preserve the pancreatic tissue on the right back and dorsal side of the bile duct and does not increase the excessive exposure of the common bile duct. During the surgery, the length of the jejunum between the pancreaticojejunostomy and the enterojejunostomy should be kept as long as possible, which is convenient for performing bileiojejunostomy as the second surgery if long-term bile duct stricture occurs. The surgical team subsequently pre-installed the common bile duct stent in patients undergoing LDPPHR and removed it one-month post-surgery. The advantage is that a hard blue-green bile duct stent can be palpated and observed during surgery, and even if the common bile duct is injured during surgery, a stage of common bile duct suture repair can be performed, albeit their effect on preventing intraoperative common bile duct injury warrants further research for validation.

The results of Chen et al. [24] showed that the operation time of LDPPHR was less than that of LPD. The average operation time of the LDPPHR group was (295 ± 42) min, and the average operation time of the LPD group was (357 ± 87) min. In this study, the average operation time of the LDPPHR group was 315 min (250 and 445 min), and the surgical time of the LPD group was 350 min (270 and 390 min), and the difference was not statistically significant. We believed that compared with LPD, LDPPHR only removes the head of the pancreas and eliminates the need for biliary-enteric anastomosis and gastrointestinal anastomosis. However, the reduction of anastomotic stoma does not mean that the operation time of LDPPHR is decreased, and LDPPHR requires complete resection of the pancreatic head. The head of the pancreas should also protect the integrity of the pancreaticoduodenal arterial arch close to the back of the pancreatic head, and the overall operation is more difficult than LPD. Notably, the LDPPHR chief surgeons in our center have undergone the LPD learning curve, and LDPPHR is implemented based on their proficiency in LPD.

Presently, the development of LDPPHR is still in the preliminary stage worldwide. The number of cases involved in this study is less, and a larger cohort and multi-centered research data are still required to verify the research results in the future. This study only performed a short-term comparative study of LDPPHR and LPD, and the corresponding long-term efficacy requires further follow-up studies.

## Conclusions

Compared with LPD, LDPPHR can decrease the postoperative exhaust time of patients, improve the short-term postoperative nutritional status, and does not decrease the safety of the perioperative period.

## Data Availability

The datasets used and/or analysed during the current study are available from the corresponding author on reasonable request.
